# Fatty acid nitroalkenes – Multi-target agents for the treatment of sickle cell disease

**DOI:** 10.1016/j.redox.2023.102941

**Published:** 2023-10-24

**Authors:** Fabliha A. Chowdhury, Nicole Colussi, Malini Sharma, Katherine C. Wood, Julia Z. Xu, Bruce A. Freeman, Francisco J. Schopfer, Adam C. Straub

**Affiliations:** a Department of Pharmacology and Chemical Biology, University of Pittsburgh, Pittsburgh, PA, USA; b Heart, Lung, Blood and Vascular Medicine Institute, University of Pittsburgh, Pittsburgh, PA, USA; c University of Pittsburgh School of Medicine, Pittsburgh, PA, USA; d Division of Hematology and Oncology, Department of Medicine, University of Pittsburgh, Pittsburgh, PA, USA; e Pittsburgh Liver Research Center (PLRC), University of Pittsburgh, Pittsburgh, PA, USA; f Center for Microvascular Research, University of Pittsburgh, Pittsburgh, PA, USA

**Keywords:** Nitro-fatty acid, Sickle cell anemia, Oxidative stress, Inflammation, Multi-organ pathophysiology, Vascular, Endothelial cell

## Abstract

Sickle cell disease (SCD) is a hereditary hematological disease with high morbidity and mortality rates worldwide. Despite being monogenic, SCD patients display a plethora of disease-associated complications including anemia, oxidative stress, sterile inflammation, vaso-occlusive crisis-related pain, and vasculopathy, all of which contribute to multiorgan dysfunction and failure. Over the past decade, numerous small molecule drugs, biologics, and gene-based interventions have been evaluated; however, only four disease-modifying drug therapies are presently FDA approved. Barriers regarding effectiveness, accessibility, affordability, tolerance, and compliance of the current polypharmacy-based disease-management approaches are challenging. As such, there is an unmet pharmacological need for safer, more efficacious, and logistically accessible treatment options for SCD patients. Herein, we evaluate the potential of small molecule nitroalkenes such as nitro-fatty acid (NO_2_-FA) as a therapy for SCD. These agents are electrophilic and exert anti-inflammatory and tissue repair effects through an ability to transiently post-translationally bind to and modify transcription factors, pro-inflammatory enzymes and cell signaling mediators. Preclinical and clinical studies affirm safety of the drug class and a murine model of SCD reveals protection against inflammation, fibrosis, and vascular dysfunction. Despite protective cardiac, renal, pulmonary, and central nervous system effects of nitroalkenes, they have not previously been considered as therapy for SCD. We highlight the pathways targeted by this drug class, which can potentially prevent the end-organ damage associated with SCD and contrast their prospective therapeutic benefits for SCD as opposed to current polypharmacy approaches.

## 1 Introduction to sickle cell disease

**Fig. 1:**
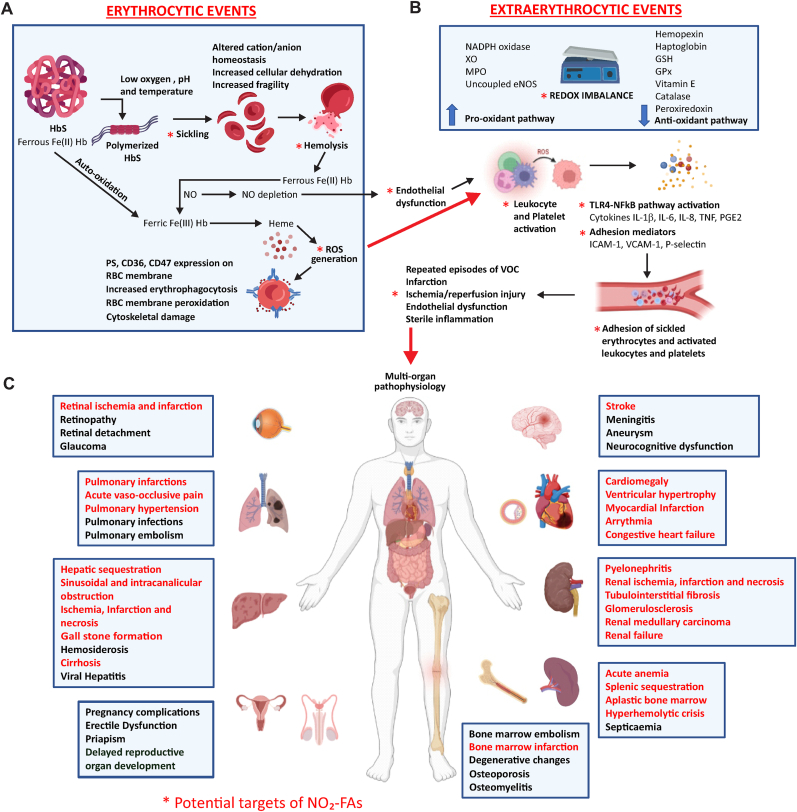
**Pathophysiological effects and consequences of SCD and the pharmacological targets of NO**_**2**_**-FA**. **1A.** Hemoglobin S (HbS) undergoes polymerization under conditions of low oxygen, pH and temperature, causing erythrocytes to sickle. The sickled erythrocytes undergo hemolysis due to cellular alterations and release hemoglobin (Hb) into the circulation. Free Hb ((Ferrous Fe (II) Hb) either undergoes auto-oxidation or reacts with nitric oxide (NO) to form methemoglobin (Ferric Fe (III) Hb). The heme supports reactive oxygen species (ROS) generation causing erythrocytic membrane peroxidation, cytoskeletal damage and phosphatidylserine (PS), CD36, and CD47-induced erythrophagocytosis. Extra-erythrocytic events follow, stimulating further ROS generation. **1B:** High levels of heme and Hb saturate hemopexin and haptoglobin, respectively. Redox imbalance occurs due to a downregulation of antioxidants: glutathione (GSH), glutathione peroxidase (GPx), vitamin E, catalase, peroxiredoxin; and upregulation of pro-oxidants: nicotinamide dinucleotide phosphate (NADPH) oxidase, xanthine oxidase (XO), myeloperoxidase (MPO), uncoupled endothelial nitric oxide synthase (eNOS). ROS cause activation of leukocytes and platelets and the Toll-like receptor 4-nuclear factor-ĸB (TLR4-NFĸB) signaling pathway. The TLR4-NFĸB pathway stimulates the release of pro-inflammatory cytokines, including Interleukin-1β (IL-1β), Interleukin-6 (IL-6), Interleukin-8 (IL-8), Tumor Necrosis Factor (TNF) and Prostaglandin E2 (PG-E_2_). Adhesion mediators including vascular cell adhesion molecule-1 (VCAM1), intracellular adhesion molecule-1 (ICAM1) and P-selectin are highly expressed on endothelium and secreted into the circulation. The cytokines and adhesion mediators promote adhesion of sickled erythrocytes and activated leukocytes and platelets to the vascular wall. These events, in addition to NO depletion, impair endothelial function and blood vessel relaxation. Blood flow encounters cellular and vascular obstacles that result in vaso-occlusive crisis (VOC). Repeated episodes of VOC become widespread across the body due to persistent oxidative stress, promoting tissue ischemia, inflammation and infarction. **1C:** Multiple end-organ injuries take place as a result, which makes SCD a multi-factorial systemic phenomenon. * and text in red indicate the SCD-associated disorders that can be targeted by **NO**_**2**_**-FA** based upon defined mechanisms of action and relevant published experimental results.

Sickle Cell Disease (SCD) is a congenital blood disorder with a worldwide incidence of 300,000 to 400,000 births annually, 75% of which prevails in Sub-Saharan Africa [1]. SCD originates from the substitution of glutamic acid by valine at the sixth amino acid position in the β-chain of adult hemoglobin (HbA) [2,3]. This results in hemoglobin S (HbS), which polymerizes in the setting of hypoxia, low pH, and decreased temperature [[2], [3], [4]]. The reduced oxygen affinity of Hb, due to elevated levels of 2,3-diphosphoglycerate, and cellular dehydration, a result of altered cation/anion homeostasis, also stimulate Hb polymerization. In aggregate, polymerized Hb fibers will sickle erythrocytes and change cell shape, flexibility, and rheology, both reversibly and irreversibly [1,5,6]. Linked with this, accelerated generation of reactive oxygen species (ROS) and iron-heme complexes generated by Hb auto-oxidation promote erythrocyte membrane lipid oxidation and cytoskeletal damage [1,5,7] (Fig. 1A). These diverse pathogenic assaults on sickled erythrocytes confer susceptibility to hemolysis, shortening their lifespan by more than 75% [1].

A toxic mélange of heme, Hb, ROS, arginase, serum lactate dehydrogenase (LDH) and microparticles containing micro-RNA, proteins, and cell surface markers are released in the circulation from hemolyzed erythrocytes, causing widespread extra-erythrocytic molecular alterations and acute oxidative stress [1,6,[8], [9], [10]]. Plasma-free heme and Hb, when in excess, can saturate their binding proteins hemopexin and haptoglobin [9]. There is also elevation of cellular nicotinamide dinucleotide phosphate (NADPH) oxidase, xanthine oxidase (XO), myeloperoxidase (MPO) and uncoupled endothelial nitric oxide synthase (eNOS) activities, which exacerbates redox imbalance [[8], [9], [10]]. This is further worsened by depletion of antioxidants such as glutathione (GSH), glutathione peroxidase (GPx), and catalase [6]. The downstream consequences of erythrocyte hemolysis impair nitric oxide (•NO)-mediated vaso-regulation and yields toxic secondary nitrogen oxides [10,11]. Hemolysis interrupts the red cell diffusion barrier for •NO, resulting in endothelial dysfunction and •NO resistance [10,12]. Free Hb in both its oxygenated (oxyhemoglobin) and deoxygenated (deoxyhemoglobin) states rapidly reacts with •NO (6–8 × 10^7^ M^−1^s^−1^); oxyhemoglobin reacts with •NO to form methemoglobin and nitrate and deoxyhemoglobin reacts with •NO to form nitrosyl Hb [[13], [14], [15], [16], [17]]. Arginine, the substrate for •NO biosynthesis, is consumed by arginase released upon hemolysis [18], and the asymmetric dimethylarginine also released by erythrocytes both conspire to further reduce eNOS activity, impairing downstream •NO signaling [10]. Collectively, this leads to endothelial, platelet and leukocyte activation, vasoconstriction, and pulmonary hypertension [10,19,20] (Fig. 1B).

This cascade of HbS-induced pathophysiological reactions contributes to vaso-occlusion, hindering microvascular blood flow and precipitating vaso-occlusive crises (VOC) manifested by ischemia, infarction, and severe pain [19,20]. Chronic oxidative stress and impaired •NO signaling stimulates endothelial cell-surface expression of the adhesion mediators vascular cell adhesion molecule-1 (VCAM-1) and intracellular adhesion molecule-1 (ICAM-1) [1,5,8], erythrocyte phosphatidylserine (PS), CD36 and CD47 and platelet P-selectin and CD40 [6]. Pro-inflammatory Toll-like receptor 4 (TLR4) and nuclear factor-ĸB (NFĸB)-regulated signaling is induced in activated endothelium [21,22], secreting coagulation factors such as von Willebrand factor (vWF) and pro-inflammatory cytokines including interleukin-1β (IL-1β), interleukin-6 (IL-6), interleukin-8 (IL-8), tumor necrosis factor (TNF) and prostaglandin E2 (PG-E_2_) [23]. These newly expressed adhesive and inflammatory mediators initiate vaso-occlusion by facilitating the adherence of sickled erythrocytes to activated endothelium and circulating activated leukocytes and platelets [5,20]. Moreover, adhesion of blood cells to the endothelium activates coagulation, further enhancing vaso-occlusion [24](Fig. 1B). Patients with SCD also have elevated platelet aggregation and thrombus formation due to the augmented levels of coagulation factors and depletion of anticoagulant proteins in plasma [[24], [25], [26]], which may underlie the increased risk of developing venous thromboembolism (VTE), especially pulmonary embolism (PE), in SCD [19,27].

The vasculopathy associated with SCD generates a multitude of acute and chronic complications in vital organs (Fig. 1C). Patients with higher Hb levels have increased frequency of pain episodes, due to an inability to compensate for the higher blood viscosity or vaso-occlusion. Patients with lower Hb levels due to severe hemolytic anemia show higher levels of free heme, serum bilirubin, and LDH [1,28]. In this case, reduced •NO levels and persistent inflammatory stress due to hemolysis and oxidative stress increase risk for further vascular complications, including pulmonary hypertension, cardiomegaly, cardio-ventricular dysfunction, ischemic stroke, leg ulcers, nephropathy, and gallstones [28]. These and other associated comorbidities shorten the life expectancy of people having SCD by an average of 30 years [29,30].

## 2 Challenges with current therapeutic management strategies

For almost two decades hydroxyurea (HU) was the only FDA-approved drug for SCD [29]. It induces fetal hemoglobin (HbF) induction 2% to over 30% [[31], [32], [33]] and reduces VOC, acute chest syndrome, infections, hospital admissions and risk for death [7,34]. Despite the benefits HU provides, adverse side effects include myelosuppression, teratogenicity, and a growing concern that long-term use of HU can lead to DNA damage, impaired spermatogenesis [35], oxidative stress, and leukemogenesis [36,37]. The considerable interpatient variability with regards to efficacy and maximum tolerated dose complicates clinical management, requiring frequent dose adjustments [38]. All these factors reduce patient adherence to HU therapy.

Blood transfusions are also frequently used as a treatment for SCD [39]. The newly transfused healthy erythrocytes dilute the percentage of erythrocytes containing HbS, improving cellular rheology in the circulation and suppressing further sickling of HbS erythrocytes [40]. However, blood transfusions can cause volume overload, iron overload, and even adverse neurological events as a result of hyperviscosity [39,41]. Although exchange transfusion can overcome these problems, the procedure is expensive and requires proper venous access (an issue in very young and old populations), skilled operators, and a high number of red cell units to reach hemoglobin goals [39,42]. Additionally, transfusions carry the risks of alloimmunization, hemolytic transfusion reactions, and hyperhemolysis in patients with SCD [36,41].

Another FDA-approved therapeutic option for SCD is hematopoietic stem cell transplantation (HSCT). This is the only clinically available curative treatment strategy for SCD, with matched related donor transplants having the best outcomes. This procedure can initiate donor-derived erythropoiesis and repair dysfunctional organs [43,44]. In addition to the significant limitations posed by donor matching, there is an increased risk for acquiring graft versus host disease (GVHD). Also, the overall complexity of the procedure and daunting economic barriers, such as a lack of sophisticated health care availability in poorer countries replete with rural areas, greatly limit widespread use of HSCT [7,29].

Four new drugs for treating SCD have received FDA approval in the last 6 years: l-glutamine, voxelotor, crizanlizumab, and deferiprone. l-glutamine is a substrate for the synthesis of nicotinamide adenine dinucleotide (NAD+) and GSH, with these metabolites viewed to improve redox state and mitigate the erythrocyte membrane damage and adhesion events that are promoted by glutamine depletion [45,46]. Voxelotor binds covalently to the N-terminal valine of the α-globin chain of HbS, where it stabilizes the oxygenated Hb state to limit the polymerization of deoxy-HbS [45,47]. Crizanlizumab is a monoclonal antibody that binds to P-selectin, thereby inhibiting the adherence of erythrocyte microparticles and activated leukocytes and platelets to the endothelium [45,48]. Clinical trial results indicate that these new therapeutic agents only partially address the complex pathophysiology of SCD. For instance, a reduced incidence of vaso-occlusion and acute chest syndrome (ACS) has been reported for crizanlizumab and l-glutamine therapy respectively, regardless of concomitant HU therapy [6,7,43,47]. There were no substantial changes in Hb, hematocrit, or reticulocyte levels [6,7,29,49]. Voxelotor increased the Hb level by a mean of 1.1 g/dl and reduced hemolytic markers, but did not reduce the frequency of VOC [7,45,47]. Shifting the Hb-oxygen affinity profile with voxelotor may have disadvantages due to impaired off-loading of bound oxygen in highly metabolic tissues where more oxygen is required [50]. Deferiprone, an oral iron chelator, is a newly approved drug for the treatment of iron overload associated with frequent blood transfusions in SCD patients [51]. Although the newly approved drugs have good safety margins, it is still unknown whether these drugs impact end-organ damage or are disease modifying [29,46].

Contemporary SCD management calls for a multi-agent therapeutic approach [1,6]; thus, combinations of prescription analgesics [52,53], antibiotics, steroids, and bronchodilators [54], along with HU and blood transfusions, are common. SCD patients require frequent doctor visits, hospital stays, blood transfusions, surgeries, and constant screening to help improve their quality of life [1,54,55]. Although advances in therapeutic management have significantly improved quality of life and the lifespan of SCD patients in high-income countries, life expectancy still lags far behind the general population. The disease burden and mortality rates for SCD worldwide are unchanged, as the poorer regions of Sub-Saharan Africa, where the majority of SCD sufferers reside [45], have restricted access to emergency care with only 19–50% of hospitals having the ability to provide 24-h medical attention and treatment [56]. It was also found that 18–41% of the facilities have expired drugs in their inventories [56]. Along with drug availability issues, the significantly lower income of many SCD patients limits access to HU and in particular newer therapeutic agents [45]. The benefits of new therapies are not cost-effective and thus disadvantageous in low-income settings [45,57,58]. Additionally, these drugs target specific features of SCD and do not have broad mechanisms of action that address the multiple pathological facets of SCD [27]. Given the current scenario, choosing expensive multi-agent treatment options which do not significantly change the course of the disease or improve the quality of life or lifespan [29,45,59], thus does not provide a rational alternative from health and economic perspectives.

## 3 The unmet clinical need for better therapeutic management of SCD

As noted, there are challenges regarding effectiveness, accessibility, affordability, tolerance, and compliance of the current polypharmacy-based approach to the therapeutic management of SCD. Herein, we detail the current state of therapeutic approaches for managing SCD in Section 2, as well as the ongoing clinical studies that aim to expand and improve future treatment options for the disease in Table 1. By doing so, our intent is to highlight the loopholes in the general concept of targeting and designing therapeutics for a multi-factorial disease like SCD.

**Table 1:** Potential therapeutic agents in pipeline for SCD.

Target/Hypothesis	Therapeutic agent	Mechanism of action	Molecular effects	Cellular effects	Organ-wide effects	Phase of Study (Reference)
HbF induction	Decitabine and Tetrahydrouridine	DNA methyl transferase inhibitor	Increase in HbF, F cells and total Hb	Increase in platelets and decrease in neutrophils and reticulocytes		Phase I NCT01685515
HbF induction	Panobinostat	Histone deacetylase inhibitor	Increase in HbF in 3 patients with Hodgkin's lymphoma	Anti-inflammatory effects		Phase I NCT01245179 [126]
HbF induction	HQK-1001	Histone deacetylase inhibitor	Increase in HbF and total Hb			Phase IIa NCT01322269
HbF induction	Pomalidomide	Lowers expression of BCL11A/SOX6	No results reported			Phase I NCT01522547
HbF induction and cellular hydration	HU and Magnesium pidolate	HU: ribonuclease reductase inhibitor and Magnesium pidolate: improves cellular hydration	No results reported			Phase I NCT00143572
Sickling of erythrocytes	SCD-101/Niprisan	HbS polymerization inhibitor			Decrease in frequency of VOC crisis and associated pain, renal and liver function remained normal	Phase I NCT02380079
				Decrease in sickling	Decrease in the frequency of VOC and associated bone pain and hospital admission; patients felt better and were more present in work in comparison to placebo	Approved in Nigeria [127]
Sickling of erythrocytes	Mitapivat (AG-348)	PKR activator	Increase in total Hb and ATP, decrease in 2,3-DPG			Phase I NCT04000165 Phase II/III NCT05031780
Sickling of erythrocytes	Etavopivat (FT-4202)	PKR activator	Increase in ATP and Hb-O_2_ affinity			Phase I NCT03815695
Sickling of erythrocytes	GBT021601	HbS polymerization inhibitor	Increase in Hb-O_2_ affinity by binding covalently to the N-terminal of the α-globin chain of HbS			Phase I NCT04983264 [128]
Sickling of erythrocytes	AES-103	HbS polymerization inhibitor	No results reported			Phase I NCT01597401
Sickling and cellular hydration	ICA-17043	Gardos channel inhibitor in erythrocytes	Increase in total Hb and decrease in LDH and indirect bilirubin	Decrease in percentage of dense erythrocytes and reticulocytes		Phase II NCT00040677
Sickling and cellular hydration	HU and clotrimazole	HU: ribonuclease reductase inhibitor and Clotrimazole: Gardos channel inhibitor in erythrocytes	No results reported			Phase I/II NCT00004492
Sickling of erythrocytes	Sanguinate	Forms Hb-CO	No results reported			Phase I NCT01848925
VOC and associated pain			No results reported			Phase II NCT02411708
VOC and associated pain			No results reported			Phase II NCT02672540
Leg ulcer			No results reported			Phase II NCT02600390
VOC and associated pain	Intravenous Immunoglobulin	Decreases interactions of blood cells and endothelium. Inhibits nucleophilic antigen Mac-1	Decrease in Mac-1 function			Phase I/II NCT01757418
VOC and associated pain	Prasugrel	ADP-receptor antagonist (P2Y12 class, anti-platelet)	Decrease in markers of platelet activation including P-selectin		Did not decrease the rate of VOC in pediatrics and adolescents up to 17 years of age	Global Phase III trial [129]
VOC and associated pain	Eptifibatide	GPIIb/IIIa inhibitor (anti-platelet)			Did not improve time to resolving VOC or hospital discharge	Phase II NCT00834899
VOC and associated pain	Low molecular mass heparin: Dalteparin	Anti-thrombin III agonist (anti-platelet)	Decrease in d-dimers from fibrin degradation and thrombin		Decrease in clinical pain scores	Phase II NCT01419977
VOC and associated pain	Low molecular mass heparin: Tinzaparin	Anti-thrombin III agonist (anti-platelet)			Decrease in frequency and duration of pain crisis and duration of hospital stay	Phase II [130]
VOC and associated pain	Ticagrelor	ADP-receptor antagonist (P2Y12 class, anti-platelet)			Did not decrease the number of pain-free days or frequency of VOC	Phase III NCT03615924
VOC and associated pain	Inhaled NO	Vasodilation	Increase in plasma nitrate		Did not decrease the time to VOC resolution	Phase II NCT00142051
VOC and associated pain	IV l-arginine	Substrate for NO synthesis		Increase in mitochondrial activity and decrease in oxidative stress	Decrease in opioid use and pain score at discharge during acute VOC	Phase II NCT02536170 NCT01796678
VOC and associated pain	Mometasone	Corticosteroid	Decrease in circulating soluble vascular cell adhesion molecule and markers of macrophage activation		Decrease in daily pain scores	Phase II NCT02061202 NCT03758950
VOC and associated pain	Sevuparin	Pan-selectin antagonist			Did not decrease to VOC resolution	Phase II NCT02515838
VOC and associated pain	IV Magnesium sulfate	Vasodilation, anti-inflammatory, pain-relief			Did not decrease length of stay, opioid use, or improve quality of life	Phase III NCT01197417
VOC and associated pain	Rivipansel	Pan-selectin inhibitor	Decrease in E-selectin		Decrease in time to discharge and time to discontinuation of opioid analgesics only if administered early	Phase III NCT02187003 NCT02433158
VOC and associated pain	Inhaled Cannabis	Analgesic			Did not decrease pain and associated symptoms	Phase I/II NCT01771731
VOC and associated pain	Poloxamer 188	Anti-inflammatory, anti-thrombotic, cyto-protective			Did not decrease opioid use and pain score at discharge during acute VOC	Phase III NCT01737814
VOC associated pain	Ketamine, Morphine	Analgesic			Intravenous ketamine provided comparable effect as intravenous morphine	Phase IV NCT02434939
VOC associated pain	Acetoamino-phen, Morphine	Analgesic			Did not decrease the requirement morphine	Phase IV NCT03541980
VOC and endothelial dysfunction	Propranolol	Anti-adhesive	Decrease in E-selectin, ICAM1, VCAM1	Decrease in adhesion		Phase II NCT01077921
VOC and tissue injury	Montelukast	Cysteinyl-leukotriene receptor antagonist	No results reported			Phase II NCT01960413
VOC and inflammation	Inhaled Mometasone	Corticosteroid	Decrease in circulating soluble vascular cell adhesion molecule and markers of macrophage activation		Decrease in daily pain scores	Phase II NCT02061202 NCT03758950
VOC and inflammation	NKTT120	Humanized anti-iNKT cell monoclonal antibody	Rapid and sustained decrease in iNKT			Phase I NCT01783691
VOC and inflammation	Zileuton	5-Lipoxygenase inhibitor			Safe and tolerable	Phase I NCT01136941
VOC and inflammation	Rivaroxaban	Factor Xa inhibitor	No changes in D-dimer, inflammatory, and endothelial activation markers or measures of microvascular blood flow			Phase II NCT02072668
VOC and inflammation	Simvastatin	HMG-CoA reductase inhibitor	Increase in plasma NO metabolites, decrease in CRP, IL-6, cholesterol		Decrease in frequency of VOC	Phase I/II NCT01702246
Oxidative stress	N-Acetyl cysteine	Antioxidant	No results reported			Phase III NCT01849016
Secretion and reactivity of VWF			No results reported			Phase I/II NCT01800526
Hematology and pain crisis	HU and l-carnitine	HU: ribonuclease reductase inhibitor and l-carnitine: antioxidant	No results reported			Phase IV NCT05081349
CRP level in pain and inflammation	Alpha-lipoic acid and l-carnitine	Antioxidant	Did not decrease CRP			Phase II NCT01054768
Oxidative stress and pain	PF-04447943	Phosphodiesterase 9 A inhibitor	Decrease in circulating E-selectin which is an adhesion mediator	Decrease in the number and size of aggregates		Phase I NCT02114203
Pain crisis and acute chest syndrome	Regadenoson	Selective A2A adenosine receptor agonist, anti-inflammatory		Decrease in percentage of inactivated natural killer T cells	Did not improve pain crisis or hospital stay	Phase II NCT01788631
Vasculopathy and pulmonary hypertension	Riociguat	Soluble guanylate cyclase stimulator	No results reported			Phase II NCT02633397
Chronic leg ulcer	Nitrites	Vasodilation, substrate for NO			Increase in blood flow to the wound, decrease in leg ulcer size and pain severity	Phase I NCT01316796
Acute chest syndrome	Varespladib	Phospholipase A2 inhibitor	No results reported			Phase II NCT00434473
Nephropathy	Losartan	Angiotensin II receptor antagonist	Decrease in urinary albumin secretion		Cardiopulmonary status remained unchanged	Phase II NCT01479439
Health related quality of life	Vitamin D				Decrease in pain, fatigue, and depression. Improvement in physical performance	Phase III NCT03417947
Efficacy and safety	IMR-687	Phosphodiesterase 9 A inhibitor	Limited data			Phase II NCT03401112

Hb: Hemoglobin, HbF: Fetal hemoglobin, Hb-CO: Carboxy-hemoglobin, VOC: Vaso-occlusive crises, CRP: C-reactive protein, LDH: Lactate Dehydrogenase, Mac-1: Macrophage-1 antigen, ICAM1: Intercellular Adhesion Molecule 1, VCAM1: Vascular Cell Adhesion Molecule 1, iNKT cells: inactivated natural killer T cells, HMG-CoA: 3-hydroxy-3-methylglutaryl coenzyme A, NO-Nitric Oxide, PKR: Pyruvate kinase R, ADP receptor: Adenosine 5'diphosphate receptor.

Table 1 lists drugs at different stages of current clinical trials for SCD and does not include the trials in recruiting phase and the upcoming gene modification therapies, as these are beyond the scope of this review. The data have been collected from some recent review articles [1,6,7,29,60,61] and clinicaltrials.gov, and organized by the targets or proposed mechanisms of each drug. The table highlights the therapeutic effect of each drug at the molecular, cellular, and organ levels. The reason behind differentiating these three levels of biological complexity is to emphasize different SCD-related actions of the drugs in pipeline. Since SCD is a systemic phenomenon, targeting a specific pathway upstream or downstream of the critical inciting pathophysiological events can attenuate modification of the disease course. The table is meant to show how a majority of the drugs in the pipeline, rather than being multi-target, are restricted to single pathological events of SCD. Consequently, most therapies do not display broadly significant clinical benefits. This highlights an unmet clinical need – the application of multi-target drugs to limit disease symptoms, crises, and progression by concomitantly modulating multiple pathways.

**Fig. 2:**
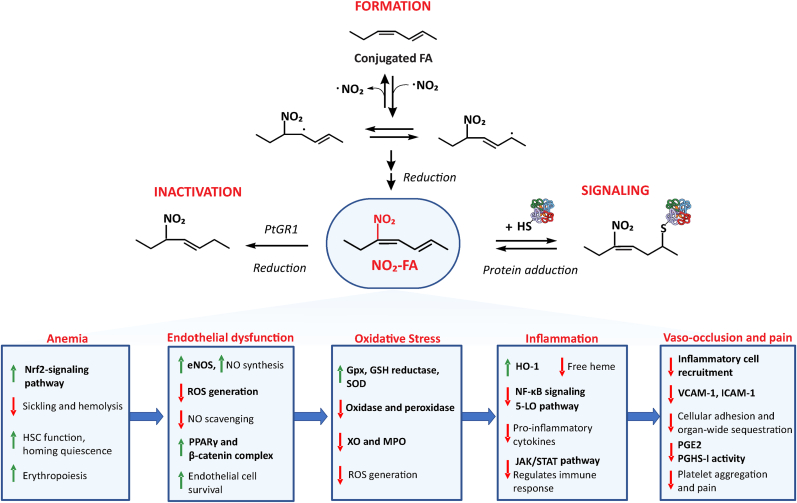
Formation, mechanism of action and potential effects of NO_2_-FA on major pathophysiological complications of SCD The endogenous formation of NO_2_-FA proceeds by the rapid addition of nitrogen dioxide to double bonds of conjugated fatty acids. Initial nitrated intermediates continue to react with oxygen and nitrogen radicals present in the reaction system but are eventually reduced to the resonance stabilized NO_2_-FA. In the free acid form, NO_2_-FA reversibly adduct to redox-sensitive cysteine residues in regulatory proteins and transcription factors. This chemical modification (i.e., nitroalkylation) introduces structural changes to the protein's native form, causing altered regulation and differential signaling responses. Important signaling pathways associated with SCD can be potentially modulated by NO_2_-FA, attenuating the major complications that cause organ-wide damage, including sickling and hemolysis, endothelial dysfunction, oxidative stress, inflammation, vaso-occlusion, and pain crises. Prostaglandin reductase 1 (PtGR-1) inactivates NO_2_-FA by disrupting its electrophilic character, thereby diminishing protein target adduction and modulation of signaling pathways. The green arrow shows induction, and the red arrow shows inhibition or down-regulation of the corresponding pathways/molecules (in bold) by NO_2_-FA. Nrf2, Nuclear factor erythroid 2-related factor; eNOS, endothelial nitric oxide synthase; ROS, reactive oxygen species; PPAR γ, peroxisome proliferator-activated receptor gamma; GPx, glutathione peroxidase; XO, xanthine oxidase; MPO, myeloperoxidase; HO-1, heme-oxygenase I; NF ĸB, nuclear factor-ĸB; JAK/STAT, Janus kinase/signal transducers and activators of transcription; 5-LO, 5-lipoxygenase; VCAM-I, vascular cell adhesion molecule-1; ICAM-I, intracellular cell adhesion molecule-1; PGE_2_, prostaglandin E2; PGHS-I, prostaglandin endoperoxide H synthase I

Here, we introduce a drug class that has not been previously tested in SCD -- electrophilic fatty acid nitroalkenes or nitro-fatty acid (NO_2_-FA). Given the multi-target actions of NO_2_-FA, we are the first to consider the suitability of the drug for targeting the multiple dysregulated pathways of SCD that lead to widespread end-organ damage: sickling and hemolysis, endothelial dysfunction, oxidative stress, inflammation, vaso-occlusion, and pain crises (Table 1). We rationalize the potential for NO_2_-FA therapy to prevent or mitigate much of the systemic pathology of SCD. Fig. 1, Fig. 2 also highlight NO_2_-FA as a prospective multi-target therapy for SCD.

## 4 Multi-target small molecule nitroalkenes as therapeutic agents for treating SCD

NO_2_-FA are endogenously generated lipid mediators normally found in plasma, urine, cell membranes and tissues in free, esterified form or adducted to nucleophilic residues in proteins [[62], [63], [64]]. NO_2_-FA are readily formed during digestion of foods such as vegetables, dairy products, and both plant and marine oils. The acidic conditions of digestion catalyzes the formation of nitrogen dioxide from nitrite, that in turn mediates the nitration of dietary unsaturated fatty acids [65]. NO_2_-FA are also formed as a consequence of inflammation, where multiple convergent reactions of superoxide, hydrogen peroxide, •NO, nitrite and heme-based catalysts ultimately give rise to the proximal unsaturated FA nitrating species, nitrogen dioxide [[62], [63], [64]].

Nitrated fatty acids have been studied both preclinically and clinically [63]. Endogenously, unsaturated fatty acids with 2 or more conjugated double bonds are the primary substrates for nitration and the endogenous generation of NO_2_-FA (Fig. 2). Thus, nitro derivatives of conjugated linoleic and linolenic acid and their metabolites are readily detectable in healthy human urine and plasma [66,67]. Naturally-occurring NO_2_-FA homologs and non-natural small molecule nitroalkenes can be synthesized by nitrosenylation or nitro-aldol condensation followed by acetylation and elimination steps, the latter approach allowing the synthesis of specific positional isomers [63]. All nitroalkenes are characterized by double bonds containing an electron-withdrawing nitro substituent that confers an electrophilic reactivity to the other carbon of the double bond. This reactivity in turn promotes Michael addition with nucleophilic (electron-rich) residues such as cysteine [68] and histidine [[68], [69], [70]]. Michael addition can also occur with lipid electrophiles having an, substituents (e.g., 4-hydroxy-2-nonenal, 4-oxo-2-nonenal, 15-deoxy-prostaglandin-J_2_) [67,71]. As a consequence of these reactions, post-translational protein modifications (PTM) occur that can trigger changes in the activity, localization, structure and function of proteins [62,72]. These PTMs, when induced by nM concentrations of reversibly-reactive small molecule electrophiles, typically promote adaptation to inflammatory, oxidative and metabolic stress [63,65](Fig. 2). NO_2_-FA impact the function of 100–150 target proteins, all having hyper-reactive cysteines, that regulate diverse signaling pathways important in regulating inflammation and vascular function. This includes nuclear factor erythroid 2-related factor (Nrf2)/KEAP1, NF-ĸB, heat shock response (HSR), peroxisome proliferator-activated receptor gamma (PPARγ), Janus kinase/signal transducers and activators of transcription (JAK/STAT), stimulator of interferon genes (STING), epoxide hydrolase, calcineurin A and angiotensin II receptor function, among others [63,64,73,74]. The protein reactions and functional impact of synthetic and endogenously occurring NO_2_-FA have been studied in multiple preclinical disease models **(reviewed in** Table 2**)**, demonstrating both anti-fibrotic and anti-inflammatory activities.

**Table 2:** NO_2_-FA evaluation in preclinical animal models.

Disease state	Animal		Disease Model	Dose	Formula-tion	Duration	Route of Admin	Outcomes
	Strain	Age						
Aortic aneurysm [132]	C57BL/6 J mice	10 wks	AngII/PCSK9 gain-of-function mutation; western diet (TD.88,137, Envigo)	5 mg/kg/day	PEG 400	4 wks	SC minipump	NO_2_-OA decreases AAA formation, inflammatory cytokine levels, and leukocyte/macrophage infiltration in the vasculature
Aortic aneurysm [133]	MFS (Fbn1C1041G/+) mice	8 wks	Genetic model on normal chow	8 mg/kg/day	PEG/ethanol (90:10, vol/vol)	4 wks	SC minipump	NO_2_-OA attenuates progression of aortic dilation in MFS via modulation of well-established disease-mediating pathways
ALS [134]	Female B6SJL-TgN (SOD1-G93A) 1Gur mice	90 days	Mouse genetic model with a G93A mutation in human SOD1	16 mg/kg 3 times per week	N/A	50 days	SC injection	NO_2_-OA improved motor performance, and reduced PGHS- and LOX derived inflammatory products in the brain.
Asthma - obese allergic airway disease [120]	C57BL/6 J mice	4 wks	HFD (60 % fat diet); 2 μg of house dust mite and cholera toxin adjuvant (0.1 μg) via orophargyneal aspiration	25 mg/kg NO_2_-OA/day	Triolein	5 days; 3 h prior to HDM challenge	PO	NO_2_-OA reduces bile acid levels by modulating hepatic expression of bile acid synthesis enzymes and reduces small airway resistance and tissue elastance
Atherosclerosis [102]	ApoE^−/−^ mice	8 wks	Atherogenic diet for 12 weeks (21 % fat and 1.25 % cholesterol)	8 mg/kg/day	PEG/ethanol	3 wks	SC minipump	Reduction of atherosclerotic lesions, inhibition of adhesion molecule expression, lower accumulation of macrophages and neutrophils in lesions.
Atrial fibrosis [135]	C57BL/6 J mice	NA	AngII infusion via mini pump (1.5 ng/g/min)	6 mg/kg/day	PEG/ethanol (90:10, vol/vol)	2 wks	SC minipump	NO_2_-OA suppressed the progression of fibrotic processes in the heart in response to Ang-II.
Atrial fibrosis and fibrillation [136]								NO_2_-OA reduces the development of atrial fibrosis and vulnerability to atrial fibrillation.
Breast cancer	Female athymic nude mice [137]	6 wks	MDA-MB-231 xenograft tumor	7.5 mg/kg/d	Sesame oil	4 wks after tumor size was 50–100 mm^3^	PO	NO_2_-OA suppressed tumor growth the mouse xenograft model.
	Female nude mice [138]			15 mg/kg/d	Tricaprylin	4 wks after tumor reached 100 mm^3^	PO	NO_2_-OA decreases the proliferation of TNBC cells, especially when co-administered with the therapeutic agents doxorubicin, cisplatin, olaparib, and gamma irradiation
Dilated cardiomyopa-thy [139]	Muscle LIM protein (MLP)-deficient mice (Mlp^−/−^)	12 wks	Genetic model on normal chow	8 mg/kg/day	PEG/ethanol (90:10, vol/vol)	4 wks	SC minipump	NO_2_-OA attenuates interstitial myocardial fibrosis and improved left ventricular systolic function in Mlp^−/−^ mice
Diabetes [140]	Lep^ob/ob^ male mice	8–10 wks	Genetic model of obesity and insulin resistance	8 mg/kg/d	N/A	4 wk	SC minipump	NO_2_-OA normalized blood glucose levels and improved glucose clearance
Hypertension	C57BL/6 J mice [141]	8–10 wks	Pharmacological model of hypertension. Ang II infusion and injection	5 mg/kg/day	N/A	2 wk	SC minipump	NO_2_-OA lowered blood pressure in response to Ang-II
				1.25, 2.5, 5, 10, 20 mg/kg		10 min before or 3 d after Ang II delivery	IV – jugular infusion	Reduced Ang II-induced hypertension independently of PPARγ activation
	C57BL/6 mice [142]	*	Ang II infusion via mini pump at 1 mg/kg/d	5 mg/kg/d		3 days after Ang II infusion	SC minipump	NO_2_-OA mediate antihypertensive signaling actions by inhibiting soluble epoxide hydrolase
Inflammation [112]	Male C57BL/6 mice	8–10 wks	Endotoxin-induced sterile sepsis	0.2 mg/kg/d	DMSO	2 days before LPS challenge	SC minipump	NO_2_-OA attenuated kidney and liver injury.
Inflammation (sepsis) [143]	C57BL/6 mice	8 wks	Cecal ligation and puncture (CLP)-induced sepsis in mice; collection of PMNs 6 h after injury	1, 10 μM	DMSO	30 min	Ex vivo PMN culture	NO_2_-OA mediated inhibition of PMNs migration is regulated by PPARγ
Inflammation (skin)	Balb/c mice, Female [144]	6–12 wks	CHS-sensitization with 0.5 % DNFB, FITC, or oxazolone	0.84 mg/kg	Ethanol; DMSO	18 h prior to skin insult	Topical	Topical NO_2_-OA treatment potentiated inflammation in CHS
	FoxP3DTR mice, Male and female [145]	6–12 wks			DMSO	18 h prior to skin insult	SC injection	NO_2_-OA inhibited skin inflammation by attenuated production of inflammatory cytokines and favored accumulation of Tregs in skin.
	C57BL/6 J mice [69]	6–8 wks	Topical dose of 62.5 mg of IMQ cream (5 % Aldara)	0.2 mg/mouse	Emulsion made of liposomes containing soybean oil, medium-chain triglycerides, and egg lecithin	18 h prior to IMQ application; every other day for 5 days	PO	NO_2_-OA downregulates the production of psoriasis-dependent inflammatory cytokines in the skin, including IL-1β, IL-23, IL-6, and IL-17
			1 μg recombinant mouse (rm)IL-23 intradermally	10 mg/kg		18 h prior to IL-23 injections; every other day for 10 days	PO	
	K14-VEGF [76]	9–10 wks	Genetic model (therapeutic)	0.2 mg/mouse		12 wks; every other day	PO	
		6–8 wks	Genetic model (preventative)	0.2 mg/mouse			PO	
	K5-IL-17C [146]	8–10 wks	Genetic model	0.2 mg/mouse		Every other day for 2 wk after disease presentation	PO	
Inflammation (vascular) [103]	C57BL/6 J mice	*	Endotoxin-induced sterile sepsis	5 mg/kg/d	N/A	3 d	SC minipump	NO_2_-OA protects against vascular inflammation by inhibition of the TLR4/NF-kB signaling.
Inflammatory Bowe Disease [147]	BALB/c mice Female	7–8 wks	Chemically induced model (2 % DSS in drinking water)	0.5 or 5 mg/kg/d	N/A	7 d	SC minipump	NO_2_-OA attenuated inflammation in experimental inflammatory bowel disease through activation of colonic PPARγ.
Ischemia reperfusion (brain) [148]	C57BL/6 J mice	8–10 wks	1 h middle cerebral artery occlusion (MCAO) + 1–7 days reperfusion	10 mg/kg	Sterile saline; mixture of 9- and 10-NO2-OA isomers (1:1)	2 h after the onset of MCAO	Tail vein injection	NO_2_-OA preserves BBB integrity and establishes neurovascular protection in ischemic brain damage
Ischemia reperfusion (heart) [105]	C57BL/6 J mice	8–12 wks	30 min unilateral ischemia, 24 h reperfusion	6.6 mg/kg	PEG/ethanol (85:15, vol/vol)	At time of reperfusion	IP	NO_2_-OA reduced infarct size, preserved left ventricular function, and reduced NFkB p65 activation.
						15 min prior to reperfusion	IP	
						3 d prior to ischemia	SC minipump	
Ischemia reperfusion (kidney) [111]	B6129SF2/J Male mice	3 mo	30 min warm, bilateral ischemia, 24 h reperfusion	0.5 mg/kg	Ethanol	Starting 1 h after ischemia, every 6 h for 24 h	IP	NO_2_-OA attenuated renal injury after I/R by reducing inflammation, PMN infiltration, and ROS generation.
Ischemia reperfusion (hindlimb) [149]	Sprague Dawley rats	2 mo	1 h unilateral ischemia, 30 min reperfusion with ultrasound-targeted cavitation	82.9 ± 5.8 nmol NO2-FA per 1 × 10^9^ Lipid nanoparticle (LNP)	LNP; 1,2-distearoyl-*sn*-glycero-3-phosphocholine, 1,2-Distearoyl-*sn*-Glycero-3-phosphoethanolamine-N-(methoxy (polyethylene glycol)-2000), and polyoxyethylene-40 stearate	3 mL/h	IV - femoral infusion	NO_2_-OA delivered locally by LNPs increases microvascular blood flow and suppresses inflammatory cytokine transcription and oxidative stress following tissue ischemia-reperfusion injury
Kidney – diabetic nephropathy [150]	LepR^db/db^ and LepR^db/m^	12 wks	Genetic model	5 mg/kg/day	Ethanol	2 wks	SC minipump	Improved renal injury in diabetic nephropathy alone or in combination with losartan.
	Sprague Dawley rats	N/A	Streptozotocin (50 mg/kg, i.p.) induced diabetes	5 mg/kg/day	N/A	Prophylactic until onset of DN or for 4 wks post onset of nephropathy	SC minipump	Prophylactic NO_2_-OA improved kidney function which was not observed for post DN treatment. NO_2_-OA reduced both serum angiotensin and PTHrP
Kidney – nephropathy [151]	BALB/c Male mice		Adriamycin-induced nephropathy;	5 mg/kg/day	Ethanol	2 d before ADR single injection	SC minipump	NO_2_-OA reduced ADR-induced nephropathy by limiting inflammation and production of reactive species.
Kidney - chronic kidney disease (CKD) [152]	129/sv Male mice	6 wks	Uninephrectomy with deoxycorticosterone supplementation	2.5 and 12.5 mg/kg/day	Sesame oil	4 wks	PO	NO_2_-OA activates anti-inflammatory and anti-fibrotic effects in the kidney and limits renal injury
Lung - fibrosis [153]	C57BL/6 J mice	6–8 wks	Intratracheal bleomycin (ITB) acute lung injury (ALI)	50 μg	10 % DMSO in PBS	At times of bleomycin instillation	IT	NO_2_-OA regulates pulmonary cell inflammatory responses to ITB-induced ALI
	C57BL/6 J Male mice	N/A	Bleomycin-induced fibrosis development in vivo; lungs were removed at 2 weeks for sectioned and precision-cut lung slices	5 μM	Ethanol; Mixture of 9- and 10-NO_2_-OA (1:1, mol/mol)	4 days	Ex vivo lung culture	NO_2_-OA inhibits activated myofibroblasts, induces dedifferentiation to fibroblasts, and reverses established lung fibrosis in murine lung slices
Lung - acute lung injury [153]	C57BL/6 J mice	6–8 wks	Hyperoxia induced acute lung injury (2 days of 95 % oxygen)	50 μg/50 μl in 10 % DMSO in PBS by IT, 25 μg/100 μl in PBS by IP	10 % DMSO in PBS	30 min before and after 48 h of hyperoxia (by IT) and 24 h after hyperoxia (by IP)	IT, IP	NO_2_-OA reduces hyperoxia-induced ALI/ARDS by regulating the antioxidant pathways and restoring the mitochondrial homeostasis by regulating mitophagy
	C57BL/6 J mice	6–8 wks	Intratracheal bleomycin (3 U/kg)	50 μg	10 % DMSO in PBS; mixture of 9- and 10-NO_2_-OA isomers	Initially with bleomycin and 72 h after insult	IT	NO_2_-OA reduced cellular infiltration, proteinaceous debris deposition, activated interstitial macrophages, and tissue injury
Lung inflammation [109]	C57BL/6 J Male mice and 5-lipoxygenase-deficient mice	8 wks	Endotoxin-induced sterile sepsis	6.6 mg/kg	50 % DMSO	1, 4 h before and 4 h after LPS	IP	NO_2_-OA protection against inflammation in septic mice is 5-LO-dependent.
NAFLD/NASH	C57BL/6 J and apoE^−/−^ Male mice [117]	8 wks	Dietary (Western and NASH diet) and genetic model	5 or 8 mg/kg/d	PEG/ethanol	12 wks	SC minipump	NO_2_-OA inhibited hepatic TAG accumulation, improved energy metabolism, and protected against NASH-diet induced liver damage.
	C57BL/6 J mice [118]	6–8 wks	Dietary model HFD	8 mg/kg/d	PEG/ethanol	6 wks	SC minipump	NO_2_-OA improved metabolic parameters, and reversed liver steatosis.
Obesity [154]	Obese Zucker rats	4 months	Genetic model	0.0075 mg/kg/d	Ethanol	2 wks	SC minipump	NO_2_-OA improved circulating lipid profiles and increased high density lipoproteins.
Parkinsons [155]	Male Lewis rats	8–9 mo	Sub-acute rotenone model of PD	5, 15, and 45 mg/kg	Mygliol 812	5 d	Oral gavage	NO_2_-OA upregulates the expression of the Nrf2 target gene HO-1 and reduces 4-HNE accumulation and formation of 4-HNE-a-synuclein adducts in the substantia nigra pars compacta (SNpc)
Pulmonary arterial hypertension	C57BL/6 J mice [78]	8–10 wks	Hypoxia (28 d at 10 % O_2_)	8 mg/kg/d	N/A	2 and 4 wks	SC minipump	NO_2_-OA protects against hypoxia-induced pulmonary hypertension
	C57BL/6 J mice [156]	6–8 wks	Dietary model (60 % HFD)	8 mg/kg/d	PEG/ethanol	6.5 wks	SC minipump	NO_2_-OA improved glucose tolerance and improved pulmonary function and reduced oxidative stress and pro-inflammatory pulmonary cytokine levels.
Total body irradiation (TBI) [157]	C57BL/6 J mice	8–10 wks	Whole-body Radiation (single sub-lethal dose of 4 Gy)	10 mg/kg	10 % DMSO in PBS; mixture of 9- and 10-NO2-OA isomers (1:1)	24 h and 30 min before irradiation; 4 and 48 h after irradiation	IP	NO_2_-OA improves the recovery of WBC and BMC and increases the granulocyte stimulating factor plasma levels in irradiated mice.
Vascular injury [82]	C57BL/6 J mice	6–8 wks	Surgical model - Wire injury of femoral artery	2 mg/kg/d	N/A	3 wks	SC minipump	NO_2_-OA inhibition of neointimal hyperplasia is HO-1-dependent.
Ventral hernia [158]	Sprague Dawley Female rats	10–12 wks	Ventral hernia rat model	∼1200 pmol/scaffold rat	Oil-water PLGA microparticle emulsion	8 wks	Scaffold delivery of NO_2_-OA	NO_2_-OA repaired the abdominal wall by improving regional angiogenesis, increasing wall thickness, and enhancing cellular infiltration.

Abbreviations: ACD, allergic contact dermatitis; ADR, adriamycin; ALS, amyotrophic lateral sclerosis; BBB, blood–brain barrier; BMC, blood mononuclear cell; CHS, contact hypersensitivity; DNFB, 1-fluoro-2,4-dinitrobenzene; DSS, dextran sodium sulfate; HFD, high-fat diet; HDM, house dust mite; HO, hyperoxia; IP, intraperitoneal; IT, intratracheal; IV, intravenous; LO, lipoxygenase; LPS, lipopolysaccharide; MI, myocardial infarction; NAFLD, nonalcoholic fatty liver disease; NASH, nonalcoholic steatohepatitis; PO, oral gavage; SC, subcutaneous; WBC, white blood cell; WD, Western diet.

**Note:** This table has been adapted [131] and the content updated to include new studies reported since 2019.

NO_2_–FAs synthesized and characterized to date include nitro-oleic acid (NO_2_-OA), nitro-linoleic acid (NO_2_-LA), nitro-arachidonic acid (NO_2_-AA), and nitro-conjugated linoleic acid (NO_2_-cLA) [62,66,67,[70], [71], [72], [73], [74], [75], [76], [77], [78], [79]]. NO_2_-OA is structurally the simplest and most studied in terms of biochemistry, signaling responses, preclinical effects, and clinical safety. The metabolism, signaling, and mechanisms of action of both endogenous and pharmacological nitroalkenes have been discussed in recent reviews [62,63]. The most studied NO_2_-OA regioisomer is 10-NO_2_-OA, with preclinical toxicology, Phase I clinical trials (n = 5) and ongoing Phase II clinical trials [67] not revealing safety concerns at therapeutic doses. In addition to the value of NO_2_-FA drug-based treatments, dietary approaches that increase the endogenous levels of NO_2_-FA have been proposed. This gains relevance as dietary supplementation of cLA (3 g) + ^15^NO_2_- (20 mg) increased plasma NO_2_-cLA in healthy volunteers to levels that parallel concentrations attained in Phase 1 clinical studies of NO_2_-OA [80,81]. cLA and ^15^NO_2_- supplementation reached similar plasma concentrations of ^15^NO_2_-cLA (C_max_ 8 nM), comparable to NO_2_-OA at the target dose defined, while conducting Phase 2 clinical trials (pulmonary arterial hypertension, chronic kidney diseases, and asthma, 150 mg dose, C_max_ 7.6 nM). These levels agree with those associated with protective pharmacological actions in murine models of disease (ranging from 5 to 30 nM) [[80], [81], [82]] [[80], [81], [82]] [[80], [81], [82]]. Overall, this highlights the potential for dietary approaches to reach pharmacological levels expected to promote beneficial actions and induce protection in SCD.

Given the multi-target reactivity of NO_2_-FA and other small molecule nitroalkenes, this class of mediators may represent an effective therapy for diseases having a multifactorial pathophysiology, such as SCD. A single drug targeting several disease-causing pathways obviates the need for multi-agent treatment regimens and potentially improving both the disease course and the quality of life for patients with SCD. Based on the mechanisms of action and pharmacological evidence collected so far for NO_2_-FA and the complex pathology of SCD, we propose that a low dose of NO_2_-FA could provide protection early on, and delay or preclude the onset of many of the pathologic manifestations of SCD. Moreover, the acute crises characteristic of SCD could be treated with higher doses of NO_2_-FA. A promising alternative might also be to use dietary supplementation of NO_2_-FA precursors (nitrite, nitrate, CLA) to achieve pharmacological levels in SCD. Of significance, and as opposed to many aldehydic or α,β-unsaturated ketone-containing fatty acid electrophiles, nitroalkene reactivity towards soft nucleophiles (predominantly cysteine) is both rapid and reversible. Thus, drug accumulation should not be dose-limiting concern [68,72]. The therapeutic potential for NO_2_-FA as a modulator of the signaling pathways associated with organ damage in SCD is described in Fig. 1, Fig. 2.

### 4.1 Erythropoiesis

SCD patients suffer from acute and chronic anemia that is precipitated by a dysfunctional bone marrow, splenic sequestration, and hemolysis of erythrocytes [29,83]. The continuous production of erythrocytes in response to the anemic stress exhausts the bone marrow, impairing erythropoiesis [83,84]. Extramedullary erythropoiesis and VOC in the spleen cause splenic sequestration and splenomegaly. During splenic sequestration, there is a drop in overall levels of Hb, circulating blood volume, erythrocytes, and platelets [83,85]. Functional hyposplenia and asplenia, due to subclinical splenic infarction, predispose to systemic infections as the spleen fails to clear encapsulated bacteria [83,85]. The Nrf2-signaling pathway enhances HSC function in the bone marrow, promotes HSC homing and quiescence, and is required for stress erythropoiesis [86,87]. Nrf2 also induces HbF synthesis that protects against erythrocyte sickling and hemolysis [[88], [89], [90]]. It has been shown that knocking out Nrf2 aggravates the pathophysiology of SCD in mice [91]. NO_2_-FA are robust activators of the transcription factor Nrf2 [63,69,71], having the potential to improve erythrocyte production and longevity, lessening the deleterious effects of hemolysis and anemia.

### 4.2 Cardiovascular disorders

Approximately 32% of SCD-associated deaths are attributed to cardiovascular events [30]. In SCD, •NO depletion causes recurrent episodes of inflammation, generation of ROS, and vaso-occlusion [5,10,24]. Persistent anemia results in cardiomegaly and left ventricular hypertrophy and dysfunction, which can lead to acute myocardial infarction, arrythmia, congestive heart failure, and death [92]. NO_2_-FA provide cardiovascular protection in ischemic heart disease, in part, by inhibiting the proinflammatory NF-ĸB signaling and impeding the transcription of pro-inflammatory cytokines [65,70]. Further vascular protection comes from the ability of NO_2_-FA to suppress ROS generation by inflammatory cells, mitochondria, and cardiomyocytes, thereby simultaneously limiting •NO consumption and the generation of secondary oxidants catalyzed by ROS and oxidase, oxygenase and peroxidase reactions [93]. In concert with the upregulation of eNOS expression, this leads to a more stable and functional vascular milieu [[94], [95], [96]].

NO_2_-FA upregulate heme-oxygenase 1 (HO-1) and heat shock protein (HSP) expression by inducing Nrf2 [97] and heat shock factor (HSF) [71] dependent responses. HO-1 catabolizes heme and hinders smooth muscle cell migration after vascular injury, inhibiting restenosis [98]. The HSPs are responsible for the proper folding of proteins in cells that become denatured under stressed conditions [71]. NO_2_-FA also increase the expression of other Nrf2-regulated genes such as GPx, glutathione reductase, and superoxide dismutase [62,99], as well as non-competitively inhibit XO [70], thereby attenuating vascular ROS generation and its sequelae. NO_2_-OA and NO_2_-LA mediate inhibition of JAK/STAT pathways and maintain vascular homeostasis by regulating the immune responses, macrophage polarization and phagocytosis, as shown in lipopolysaccharide (LPS) injected murine models [100,101]. NO_2_-OA also lowers the expression of adhesion mediators, limiting vascular infiltration of inflammatory cells [102] and inhibiting venular leukocyte sequestration [103]. Finally, NO_2_-AA inhibits platelet aggregation via irreversible inactivation of prostaglandin endoperoxide H synthase I (PGHS-I) [104].

These protective effects of NO_2_-FA are of relevance to cardiac function as well, including: reduced ischemic heart injury with low neutrophil accumulation and MPO in the infarct zone [105]; blunted cardiac remodeling with limited matrix-metalloproteinase activity [66]; decreased risk for acute ventricular tachycardia (VT) with homeostatic regulation of calcium [75] and attenuation of myocardial infarct-induced cardiac hypertrophy with NO_2_-cLA and nitrite therapy [106]. These multi-target actions of NO_2_-FA have the potential to significantly impact cardiovascular function and inflammatory stress in SCD patients.

### 4.3 Pulmonary complications

Pulmonary disorders are responsible for 28% of mortality in SCD [30], with ACS and pulmonary hypertension (PH) being the most common [83,107]. ACS can arise from pulmonary infections and edema, pulmonary embolism and infarction, pulmonary vaso-occlusion, and fat emboli from the bone marrow [92,108], all manifested by pulmonary infiltrates accompanied by chest pain, fever, tachypnea, wheeze, and cough. Pulmonary hypertension can develop from an altered immune response, endothelial dysfunction, vascular bed damage, parenchymal fibrosis, and smooth muscle hypertrophy [83,92]. The ability of NO_2_-FA to protect against PH has been shown in several studies. NO_2_-OA and NO_2_-LA reduced PH in LPS-treated mice by depleting circulatory and pulmonary levels of 5-lipoxygenase (5-LO) and its downstream products leukotriene B4 (LTB4), 5-hydroxyeicosatetraenoic acid (5-HETE) and 12-HETE [109]. In an insulin-resistance model, treatment with NO_2_-FA improved PH by reducing ROS, XO, and cytokine levels [70]. NO_2_-FA administration also increases the survival of lung endothelial cells by promoting PPARγ and β-catenin complexation and upregulation of pro-survival molecules such as apelin in the endothelium [110]. 10-NO_2_-OA also inhibits transforming growth factor-β (TGF-β) signaling, attenuating vascular fibrosis and right ventricular pressure [78]. Thus, there are several avenues by which NO_2_-FA may protect against pulmonary complications traditionally associated with SCD.

### 4.4 Renal and hepatobiliary complications

Renal failure due to vaso-occlusion-induced ischemia, infarction, hyperfiltration, glomerulosclerosis, tubular injury, and necrosis is common in SCD [83] and contributes to 16% of deaths [30]. Early treatment with NO_2_-FA modifies the inflammatory state associated with renal disease and organ failure. In nephropathic mouse models, NO_2_-OA reduces tubulointerstitial fibrosis, glomerulosclerosis, oxidative stress, and renal inflammation [76,111]. In a multi-organ endotoxemia model, inflammatory markers including TNFα, ICAM-1, VCAM-1 and PGE_2_ were reduced after treatment with NO_2_-OA [112]. Moreover, NO_2_-OA reduces ischemic injury to mouse kidneys by reducing renal MPO levels [111]. NO_2_-FA also antagonize inflammation-induced carcinogenesis by modulating oxidative stress via inhibition of NFĸB and 5-LO pathways [62], a mechanism relevant to renal medullary carcinoma. This disorder is rare, but when associated with SCD and sickle cell trait its course is extremely aggressive in the young [83,113,114].

In the liver, acute VOC results in hepatic sequestration, sinusoidal obstruction, intracanalicular cholestasis, ischemia, and necrosis [83,115], all of which may be limited by the anti-inflammatory and antioxidant activities that are instigated by downstream NO_2_-FA signaling. Common SCD-associated hepato-pathologies include intrasinusoidal sickling and dilation, erythrophagocytosis with Kupffer cell hyperplasia, cirrhosis, and hemosiderosis. Multiple nitroalkenes are effective activators of Nrf2-mediated HbF synthesis, which can blunt erythrocyte sickling and hemolysis [[88], [89], [90]]. NO_2_-FA can also limit CD36-mediated erythrophagocytosis [6,62]. In SCD, long-term hepato-fibrosis resulting from oxidative injury leads to liver cirrhosis [116], a process that NO_2_-FA can counteract and even reverse [117]. NO_2_-OA treatment of a murine non-alcoholic fatty liver disease (NAFLD) model improved energy metabolism in concert with blocking steatosis and fibrosis [118]. Lastly, in the gallbladder, the chronic hemolysis associated with SCD leads to gallstone formation and bile-stained infarcts [115,119]. NO_2_-FA also favorably regulate bile acid biosynthetic enzyme expression via upregulation of hepatic farnesoid X receptor levels [120].

### 4.5 Other tissues and organs

SCD also affects the nervous, musculoskeletal, and ophthalmic systems [29,30,83]. Neuro-vascular occlusion, caused by sickling, hemolysis, and inflammation, promotes ischemic stroke and silent cerebral infarction, particularly in children. For adults with SCD, hemorrhagic strokes may be more common [121,122]. NO_2_-FA have neuroprotective activity, as demonstrated by PPARγ activation [62] and regulation of nociceptive neuronal cells that control inflammation and pain in the central nervous system [123,124]. Osteo-vascular and retino-vascular occlusion also occur, with the former provoking infarct, infection, necrosis, and degeneration [83] and the latter causing retinopathy and blindness via peripheral retinal ischemia [125]. To date, the potential therapeutic effects of NO_2_-FA in osteopathy and visual systems have yet to be explored.

## 5 Conclusions

In the last few decades, remarkable advances have been made in discerning the pathophysiological mechanisms of SCD. A plethora of therapeutic agents have been proposed and tested to combat this hereditary hematological disorder [6,7]. However, only four new disease-modifying agents have FDA approval, and these agents do not replace conventional therapeutic regimens in terms of cost-benefit ratio and effectiveness [45]. It is possible that current drugs in the development pipeline may also fall short of significant therapeutic responses, due to an inability to limit the multiple ongoing pathophysiological insults in SCD. NO_2_-FA, being Michael acceptors, appear to modulate the most significant pro-inflammatory reactions and signaling pathways associated with the complex pathophysiology of SCD [62,63,70,73]. This unique multi-target property suggests that NO_2_-FA could be efficacious in treating not only the symptoms and end organ damage of SCD but also other hemoglobinopathies. It is noteworthy that preclinical studies coming from multiple labs show NO_2_-FA are proficient in treating cardiovascular, pulmonary, renal, and hepatobiliary complications – the leading causes of death in SCD [30]. Hence, disease modifying opportunities can stem from developing specific nitroalkenes screened to optimize the targeting of both the causes and consequences of SCD. This would involve tracking screening and efficacy criteria that includes the induction of HbF, the activation of key protective signaling pathways and the limitation of inflammation, thus promoting a significant improvement in the morbidity and mortality of this multi-systemic disorder that affects millions of individuals worldwide.

## Sources of funding

We thank the 10.13039/100007921University of Pittsburgh and the 10.13039/100008227ARCS foundation for their continued support. Financial support for this work was provided by the 10.13039/100000002National Institutes of Health grants: R35 HL161177 (A C. Straub), R01 HL 149825 (A C. Straub), R01 HL 153532 (A C. Straub), 10.13039/100000968American Heart Association grants: Established Investigator Award 19EIA34770095 (A C. Straub), R01 GM125944 (F.J. Schopfer), R33 HL157609, (B.A. Freeman) and R01 HL162787 (B A. Freeman). This work was also supported in part by the 10.13039/100000002National Institutes of Health Grant 5KL2TR001856-07.

## Declaration of competing interest

The authors declare that Dr. Straub received research funds from Bayer Pharmaceuticals and has an interest in Creegh Pharmaceuticals. Dr. Schopfer has an interest in Creegh Pharmaceuticals, Inc and Furanica, Inc. Dr. Freeman has an interest in Creegh Pharmaceuticals, Inc. and Brainstage, Inc. Dr. Xu received research funds from and served on an advisory committee for GlaxoSmithKline and is the US national principal investigator for the Phase 1 clinical trial of AG-946 in patients with sickle cell disease.

## Data Availability

No data was used for the research described in the article.
